# Knockdown of autophagy-related gene LC3 enhances the sensitivity of HepG_2_ cells to epirubicin

**DOI:** 10.3892/etm.2015.2266

**Published:** 2015-02-05

**Authors:** WANXIN PENG, TONG DU, ZIHAO ZHANG, FENGYI DU, JIE JIN, AIHUA GONG

**Affiliations:** School of Medical Sciences and Laboratory Medicine, Jiangsu University, Zhenjiang, Jiangsu 212013, P.R. China

**Keywords:** hepatocellular carcinoma, lentivirus, autophagy, light chain 3, chemoresistance

## Abstract

Hepatocellular carcinoma (HCC) is a major public health problem. Despite new chemotherapeutic treatments, drug resistance remains a major clinical obstacle to successful treatment in HCC patients. Therefore, novel therapeutic targets and new modalities of treatment are urgently required. In this study, tetracycline-inducible lentivirus-mediated RNA interference (RNAi) was employed to knock down microtubule-associated protein 1 light chain 3 (LC3) gene, which encodes a key protein in the induction of autophagy, to study the protective function of autophagy in liver cancer tolerant to epirubicin. The effect of combined treatment with lentiviral shLC3 and epirubicin on cell growth and chemosensitivity to epirubicin in the HCC cell line HepG_2_ were also investigated. The results demonstrated that lentivirus-mediated LC3 silencing significantly inhibited cell proliferation. In addition, combined treatment with lentiviral shLC3 and epirubicin significantly decreased the survival rate of HepG_2_ cells, compared with that following treatment with either agent alone. Overall, the results from this study suggest for the first time, to the best of our knowledge, that LC3 plays a key role in HCC tumorigenesis, and is a novel therapeutic target for HCC.

## Introduction

Hepatocellular carcinoma (HCC) is the fifth most prevalent type of cancer and the third most common cause of cancer-associated mortality worldwide. Each year there are ~630,000 new cases of HCC, more than half of which occur in China (1). Despite recent advances in the understanding of the molecular basis of HCC and new therapeutic approaches, the mortality rate has declined only modestly. Drug resistance remains a major clinical obstacle to successful treatment in HCC patients (2).

Autophagy (which means self-eating) is a dynamic process in which subcellular membranes are rearranged to sequester cytoplasmic constituents, including organelles, for delivery to a lysosome or vacuole where the sequestered cargo is degraded and recycled (3). The formation of autophagosomes, double-membrane vesicles that deliver the cytoplasmic material, is central to this process. Autophagosome formation involves the conjugation of cytosolic microtubule-associated protein light chain 3 (LC3-I) with phosphatidylethanolamine to form LC3-phosphatidylethanolamine (LC3-II) as an essential process. Therefore, the ratio of LC3-II and LC3-I levels can be used as a marker to reflect the activation of autophagy (4,5).

During periods of nutrient deprivation, autophagy degrades intracellular proteins to serve as substrates for ATP generation (6). Autophagy is essential during starvation, cellular differentiation, cell death and aging, and also in the prevention of certain types of cancer and increased tumor cell survival (7,8). Autophagy induction (autophagic activity above basal levels) may occur with anticancer drug treatment as an adaptive response and can lead to chemoresistance (9). For example, when exposed to low doses of radiation, breast, colon and prostate carcinoma cells accumulate acidic vesicular organelles (AVOs) due to autophagy; this is a defense mechanism that increases the survival of irradiated cells (10). The p53-induced transcription of proteins involved in the positive regulation of the autophagy pathway is the common mechanism by which different types of anticancer drugs such as DNA-damaging agents, microtubule interfering molecules, and kinase inhibitors trigger autophagy (11,12).

Defects of autophagy are associated with numerous diseases and tumors (13,14). However, only a few studies have examined hepatocellular carcinoma (HCC) in relation to these processes (15,16). Therefore, in the present study, inducible lentivirus-mediated RNA interference (RNAi) of LC3 was used to investigate the association between autophagy and chemoresistance in HCC. The aim of the study was to improve our understanding of autophagy in human liver cancers and delineate the possible role of autophagy as a novel target for anticancer therapy.

## Materials and methods

### Cell culture and treatment conditions

The human hepatoma cell line HepG_2_ was purchased from the Cancer Cell Repository (Shanghai Cell Bank, Shanghai, China) and maintained in Dulbecco’s modified Eagle’s medium (high glucose; Gibco Life Technologies, Carlsbad, CA, USA) and supplemented with 10% fetal bovine serum (FBS; Gibco Life Technologies) in a humidified incubator with 95% air and 5% CO_2_ at 37°C. DMEM without serum was also used in certain experiments. All small hairpin RNA (shRNA)-expressing stable cell lines were grown in DMEM supplemented with 10% tetracycline (Tet)-approved FBS (Clontech Laboratories, Inc., Mountain View, CA, USA) and 1 μg/ml puromycin (Gibco Life Technologies).

### Antibodies and agents

The anti-LC3B rabbit polyclonal antibody used in the western blot assay was from Cell Signaling Technology (#12741S; Beverly, MA, USA), the anti-p62/sequestosome 1 (SQSTM1) rabbit polyclonal antibody was obtained from Proteintech (#18420-1-AP; Chicago, IL, USA) and the anti-GAPDH mouse monoclonal antibody was from Beijing CoWin Biotech Co., Ltd. (cw0100A; Beijing, China). Goat anti-rabbit immunoglobulin G-horseradish peroxidase (IgG-HRP) and goat anti-mouse IgG-HRP (Pierce, Thermo Fisher Scientific, Rockford, IL, USA) were used as secondary antibodies, and enhanced chemiluminescence reagent (Pierce, Thermo Fisher Scientific) was used for developing the blots. The agents epirubicin, doxycycline, 3-methyladenine (3-MA; Sigma-Aldrich, St. Louis, MO, USA) and purimycin (Invitrogen Life Technologies, Carlsbad, CA, USA) were dissolved in phosphate-buffered saline (PBS) to form stock solutions and then added directly into the media to the required concentration.

### Inducible lentiviral shRNA constructs

To generate shRNA-expressing plasmids, the double-stranded oligonucleotides encoding the desired shRNA were cloned into the *Age*I and *Eco*RI restriction sites of pLKO-Tet-On vector (Addgene, Inc., Cambridge, MA, USA) as described previously (17). The constructs containing an LC3-specific shRNA sequence 5′-CTGAGATCGATCAGTTCAT-3′; and scrambled shRNA 5′-GCAAGCTGACCCTGAAGTTCAT-3′ were designated pLKO-Tet-shLC3 and pLKO-Tet-shCon, respectively.

### Virus production and production of stable cell lines

Lentiviruses were generated by co-transfecting 293T cells with 1.5 μg shRNA-encoding plasmid and 1 μg pPAX2 and 0.5 μg pDMG2 (Addgene, Inc.) as helper plasmids, using Lipofectamine 3000 reagent (Invitrogen Life Technologies) according to the manufacturer’s instructions. Growth media was exchanged after 8–16 h and lentivirus-containing supernatant was harvested 24, 48 and 72 h later.

For target cell transduction, HepG_2_ cells were passaged to 40% confluency the following day. Viral medium was added to the cells with 8 μg/ml Polybrene (Sigma-Aldrich). After 24 h, viral particle-containing medium was removed and replaced with fresh medium containing 1 μg/ml puromycin. From days 4 to 10, fresh medium was replaced when necessary and evaluated for cytotoxicity under a microscope. Finally, the cells, named HepG_2_^shCon^ and HepG_2_^shLC3^, were collected for further experiments.

### Immunoblot analysis

Cells were trypsinized, washed with ice-cold PBS and lysed with RIPA buffer (50 mM Tris, 150 mM NaCl, 1% NP-40, 0.5% deoxycholic acid and 0.1% SDS), Protein protease inhibitor mixture (Thermo Fisher Scientific) was added prior to extraction. The lysates were fractionated on an SDS-PAGE gel and transferred onto a Trans-Blot polyvinylidene difluoride membrane (Bio-Rad Laboratories, Inc., Hercules, CA, USA). Blots were blocked with 5% non-fat dry milk followed by probing with the LC3B (1:1,000 dilution, CST) and p62 (1:1,000 dilution, Proteintech) primary antibodies and GAPDH (1:10,000 dilution, CoWin) overnight at 4°C. The membranes were then washed thrice with tyrosylprotein sulfotransferase, incubated with secondary antibodies at room temperature for 2 h and subsequently washed a further three times for developing. The corresponding bands were detected using the Pierce Western HRP protocol.

### Reverse transcription-quantitative polymerase chain reaction (RT-qPCR)

RNA was isolated with TRIzol reagent (Invitrogen Life Technologies), treated with DNAse (Promega Corporation, Madison, WI, USA), and then first strand cDNA was created with M-MLV reverse transcriptase (Promega Corporation) according to the manufacturer’s instructions. qPCR was performed in triplicate in 20-μl reactions with iQ SYBR^®^ Premix Ex Taq™ Perfect Real Time (Bio-Rad Laboratories, Inc.), 50 ng first strand cDNA and 0.2 μg each LC3 primer: Forward, 5′-GAGAAGCAGCTTCCTGTTCTGG-3′ and reverse, 5′-GTGTCCGTTCACCAACAGGAAG-3′; or 0.2 g each GAPDH primer: Forward, 5′-GGGTGTGAACCATGAGAAGT-3′ and reverse, 5′-GTAGAGGCAGGGATGATGTT-3′. Samples were cycled once at 95°C for 2 min, then subjected to 40 cycles of 95°C, 58°C and 72°C for 30 sec each. The relative LC3 mRNA content was calculated using the 2^−ΔΔCT^ method with GAPDH as an endogenous control.

### Detection of AVOs with acridine orange (AO) staining

Autophagy is characterized by the formation of AVOs. Cells were stained with AO (Sigma-Aldrich) as described previously (18). Briefly, in AO-stained cells, the cytoplasm and nucleolus fluoresce bright green and dim red, respectively, whereas AVOs fluoresce bright red. HepG_2_^shCon^ and HepG_2_^shLC3^ cells were cultured with or without doxycycline (10 μg/ml) for 24 h, then cultured with serum-free DMEM for 2 h. AO was then added at a final concentration of 1 μg/ml for a period of 15 min. The cells were washed with PBS 3 times, and the AVOs were visualized using a fluorescence microscope (Leica DMR, Leica Microsystems GmbH, Wetzlar, Germany) or quantified by flow cytometry (FACScan system; BD Biosciences, San Jose, CA, USA.

### Determination of the mean red:green fluorescence ratio in AO-stained cells using flow cytometry

The intensity of the red fluorescence is proportional to the degree of acidity and/or the volume of the cellular acidic compartment. Thus, by comparing the mean red:green fluorescence ratio of different cell populations, the change in the degree of acidity and/or the fractional volume of their cellular acidic compartment was measured. Cells were stained with AO for 15 min, removed from the plate with trypsin-EDTA and collected in phenol red-free growth medium. Green (510–530 nm) and red (>650 nm) fluorescence emissions from 1×10^4^ cells, illuminated with blue (488 nm) excitation light, were measured using a FACScan system and CellQuest software (BD Biosciences).

### Cytotoxicity assay

Chemotherapy drug cytotoxicity was assessed *in vitro* using the Cell Counting kit (CCK)-8 assay (7Sea Biotech, Shanghai, China) as described previously (19). In brief, 2×10^3^ cells were seeded in a 96-well flat-bottomed plate, grown at 37°C for 24 h, and then placed in doxycycline (10 μg/ml). Subsequently, cells were treated with epirubicin at increasing concentrations. After 72 h of culture, 10 μl CCK-8 reagent was added to each well and the plate was incubated for 1 h. Absorbance was read at 450 nm. All samples were carried out in sextuplicate. Data are represented as the percentage reduction in metabolic activity, normalized to HepG_2_^shCon^ cells.

### Cell cycle analysis

Following treatments, cells were trypsinized, washed with PBS and fixed in ice cold 75% ethanol. Cells were then washed with PBS and stained with propidium iodide (Invitrogen Life Technologies) and analyzed by flow cytometry on a FACScan system. Cell cycle distribution was analyzed using CellQuest software.

### Statistical analysis

Statistical analysis was performed using GraphPad software (GraphPad Software, Inc., La Jolla, CA, USA). All quantitative data are expressed as the mean ± standard deviation. Statistical differences between groups were compared using a Student’s t-test. P<0.05 was considered to indicate a statistically significant result.

## Results

### Inducible downregulation of LC3 expression in HepG_2_ cells

**Figure 1:**
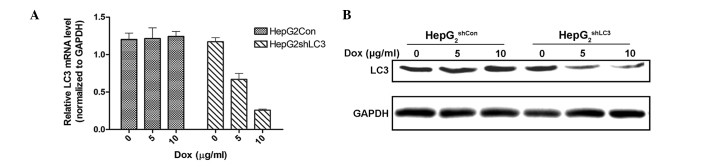
Efficient suppression of LC3 by lentiviral shRNA in HepG_2_ cells. HepG_2_ cells were treated with different concentration of Dox for 48 h. (A) Reverse transcription-quantitative polymerase chain reaction showing efficient inducible knockdown of LC3 mRNA in HepG_2_^shLC3^ cells.(B) Reduction of LC3 protein revealed by western blotting in HepG_2_^shLC3^ cells. sh, small hairpin; LC3, microtubule-associated protein 1 light chain 3; Con, control; Dox, doxycyline.

To assess the knockdown of LC3 expression, HepG_2_^shCon^ and HepG_2_^shLC3^ cells were cultured in the presence of increasing amounts of doxycycline (0, 5 and 10 μg/ml) for 72 h and analyzed by immunoblotting and RT-qPCR. In the absence of doxycycline, LC3 protein levels did not differ between the two types of cells. Increasing concentrations of doxycycline, however, resulted in the effective downregulation of LC3 expression in the HepG_2_^shLC3^ cells (Fig. 1).

### Knockdown of LC3 inhibits serum deprivation-induced autophagy in HepG_2_ cells

**Figure 2:**
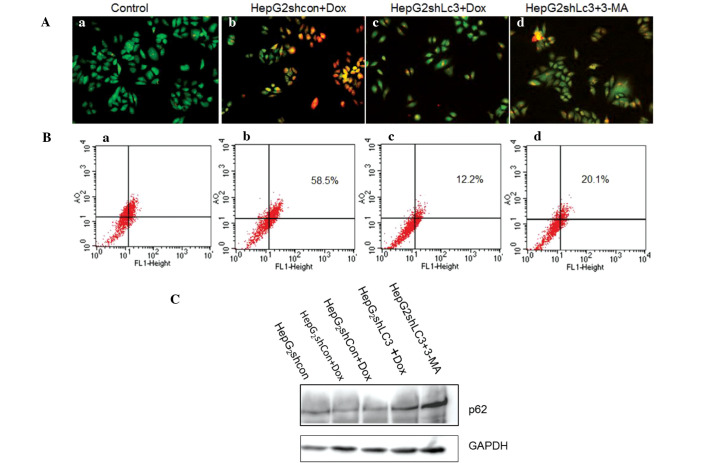
Detection of serum starvation-induced AVOs by staining with acridine orange. Cells were exposed to the supravital stain acridine orange 2 h after treatment with serum starvation. (A) Fluorescent images of AVOs (magnification, ×20): (a) untreated cells; (b) and (c) HepG_2_^shCon^ and HepG^shLC3^ cell pre-cultured with 10 μg/ml Dox, then treated with serum-free medium for 2 h. (d) HepG_2_^shLC3^ cells incubated with 5 mM 3-MA for 12 h before serum deprivation (positive control). (B) Determination of the mean red:green fluorescence ratio in acridine orange-stained cells by flow cytometry. The mean red:green fluorescence ratio was determined as described in Materials and methods. (a) Untreated cells; (b) and (c) HepG_2_^shCon^ and HepG^shLC3^ cell pre-cultured with 10 μg/ml Dox, then treated with serum-free medium for 2 h. (d) HepG_2_^shLC3^ cells incubated with 5 mM 3-MA for 12 h before serum deprivation (positive control). (C) Detection of p62 expression. Lane 1, untreated cells; lanes 2–4, HepG_2_^shCon^ and HepG^shLC3^ cells pre-cultured with 10 μg/ml Dox, then treated with serum-free medium for 2 h; lane 5, HepG_2_^shLC3^ cells incubated with 5 mM 3-MA for 12 h before serum deprivation (positive control). AVOs, acidic vesicular organelles; sh, small hairpin; LC3, microtubule-associated protein 1 light chain 3; Con, control; Dox, doxycyline; 3-MA, 3-methyladenine.

As LC3 has been shown to control the initiation of autophagy, the effect of LC3 inhibition on serum starvation-induced autophagy in HepG_2_ cells was assessed. AO staining was used to reveal AVOs following serum starvation treatment by fluorescence microscopy and FACS scanning. The data (Fig. 2A and B) demonstrate that the inhibition of LC3 resulted in a marked inhibition of the total number of autophagosomes; the 5 mM 3-MA group served as a positive control. The p62 protein, also known as SQSTM1, is itself degraded by autophagy and may serve to link ubiquitinated proteins to the autophagic machinery to enable their degradation in the lysosome. Therefore, the induction of autophagy after 2 h serum-starvation was assessed using immunoblotting to detect the expression level of p62, with GAPDH as the loading control (Fig. 2C). The degradation of p62 was repressed in the Dox (10 ug/ml) and 3-MA groups. These results indicate that the knockdown of LC3 by inducible shRNA may inhibit starvation-induced autophagy.

### Knockdown of LC3 significantly causes cell cycle arrest in HepG_2_ cells

**Figure 3:**
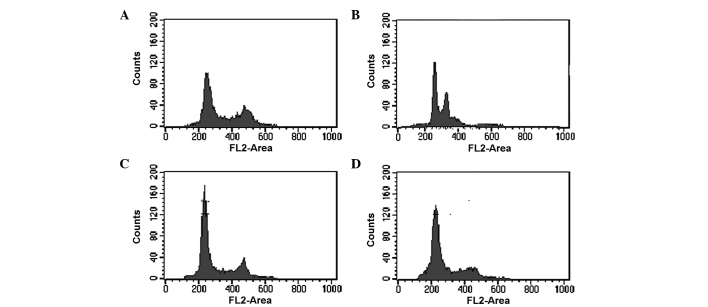
LC3 inhibition improved the epirubicin-induced cell cycle arrest of HepG_2_ cells. Following LC3 inhibition with 10 μg/ml Dox, cells were treated with epirubicin (4 μM) for 24 h, then stained with PI and analyzed using flow cytometry. Cell cycle analysis revealed that genetic and chemical inhibition of autophagy caused a significant increase of the cell population in the G1 phase of the cell cycle. (A) Untreated cells; (B) and (C) HepG_2_^shCon^ and HepG^shLC3^ cells cultured with 10 μg/ml Dox and epirubicin for 24 h. (D) HepG_2_^shLC3^ cells incubated with 5 mM 3-MA for 12 h before epirubicin treatment. Dox, doxycycline; PI, propidium iodide; sh, small hairpin; Con, control; LC3, microtubule-associated protein 1 light chain 3; 3-MA, 3-methyladenine.

Epirubicin is a widely used anthracycline drug for chemotherapy that targets DNA by intercalating DNA strands and inhibits DNA and RNA synthesis. The effect of LC3 inhibition on epirubicin-induced cell cycle abnormalities in HepG_2_ cells was investigated using 5 mM 3-MA treated group as the positive control (Fig. 3). The results indicated that knockdown of LC3 increased the percentage of G1 (2N DNA content) phase cells (Fig. 3C) compared with the shCon group (Fig. 3B) following epirubin treatment. The 3-MA group exhibited a similar result (Fig. 3D) to the shLC3 group.

### Silencing of LC3 expression sensitizes HepG_2_ cells to the therapeutic effect of epirubicin

**Figure 4:**
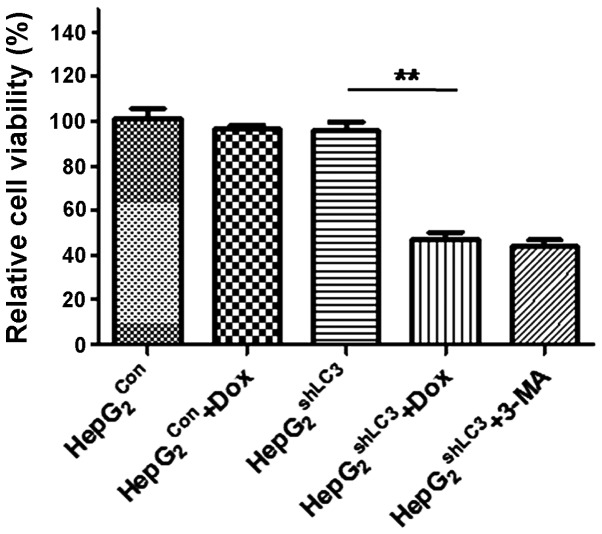
Knockdown of LC3 increased the sensitivity of HepG_2_ cells to chemotherapeutics. The CCK-8 assay showed that knockdown of LC3 by 10 μg/ml Dox had a synergistic effect with epirubicin on cell survival and proliferation. Results are expressed as the relative % live cells compared with untreated HepG_2_^shCon^ cells. Data shown are the mean + standard error of the mean (n=6). ^**^P<0.01. sh, small hairpin; Con, control; LC3, microtubule-associated protein 1 light chain 3; CCK, cell counting kit; Dox, doxycycline.

As autophagy normally promotes the resistance of tumor cells to chemotherapy, whether knockdown of LC3 increases the sensitivity of tumor cells to chemotherapy was investigated. HepG_2_^shCon^ and HepG_2_^shLC3^ cells were treated with epirubicin. The effects of LC3 knockdown with and without epirubicin on cell viability were assessed by CCK-8 assays. LC3 knockdown (induced by 10 μg/ml doxycycline) combined with epirubicin (4 μM) treatment decrease the viability of HepG_2_^shLC3^ by ~50% (P<0.01) compared with those of HepG_2_^shLC3^ cells treated with epirubicin and HepG_2_^shCon^ (Fig. 4).

## Discussion

HCC is one of the most prevalent cancers worldwide, accounting for 85–90% of all primary liver cancers, which represents ~4% of all newly diagnosed cancer cases (20). Although cytotoxic chemotherapy has been used for >30 years, definite evidence that it prolongs survival has been lacking (21). Various treatment options are available for patients with HCC according to the degree of background liver damage, tumor diameter and other factors associated with disease progression (22). Resistance to chemotherapy drugs remains a significant barrier for cytotoxic agents, often leading to chemotherapy failure in patients with HCC. Understanding of the underlying mechanisms is critical if outcomes are to be improved. There are numerous putative mechanisms for the process by which chemoresistance is induced (23–25).

Autophagy was first observed in yeast as a survival mechanism when nutrients were limited, and exists in all eukaryotic cells from yeast to mammals (26). Autophagy is a regulated lysosomal pathway for the degradation and recycling of long-lived proteins and organelles. Nutrient starvation is the most commonly used method for inducing autophagy (27). In autophagy, LC3, a mammalian homolog of yeast ATG8, is activated and relocalizes to intracellular vesicles when the lipid bilayer structure sequesters cytoplasm to form autophagosomes. Firstly, LC3 pro-form is cleaved to form soluble LC3-I. This is then modified to a membrane-bound form, known as LC3-II, which is recruited onto the autophagosomes (28,29). Autophagy occurs in response to various other forms of stress, including oxygen or growth factor deprivation and chemotherapeutics (30,31). Recent evidence suggests that autophagy provides a protective function in tumor cells in response to metabolic stress. A number of studies have reported that autophagy is activated in cancer cells in response to various anticancer therapies (32–34). However, little is known about the role of autophagy in HCC chemo-resistance. Epirubicin is a structural analog of doxorubicin that is commonly used to treat HCC. It is usually better tolerated compared with doxorubicin in the treatment of HCC (35).

In the present study, epirubicin-activated autophagy was shown to be inhibited by LC3 RNAi. The observed chemoresistance was found to be associated with the epirubicin-induced activation of autophagy-associated signaling in HepG_2_ cells. The results show that autophagy can be significantly inhibited by lentivirus-mediated RNAi of LC3 resulting in enhanced cell sensitivity to chemotherapy. These findings suggest that the knockdown of LC3 offers a novel approach that increases the sensitivity of tumor cells to chemotherapeutic agents that target DNA. In addition, they indicate that epirubicin induced autophagy as a pro-survival mechanism in HCC, which caused the HCC cells to be resistant to anthracyclines.
